# Developing Transgenic *Jatropha* Using the *SbNHX1* Gene from an Extreme Halophyte for Cultivation in Saline Wasteland

**DOI:** 10.1371/journal.pone.0071136

**Published:** 2013-08-05

**Authors:** Bhavanath Jha, Avinash Mishra, Anupama Jha, Mukul Joshi

**Affiliations:** Discipline of Marine Biotechnology and Ecology, CSIR-Central Salt and Marine Chemicals Research Institute, Bhavnagar, Gujarat, India; Virginia Tech, United States of America

## Abstract

*Jatropha* is an important second-generation biofuel plant. Salinity is a major factor adversely impacting the growth and yield of several plants including *Jatropha*. *SbNHX1* is a vacuolar Na^+^/H^+^ antiporter gene that compartmentalises excess Na^+^ ions into the vacuole and maintains ion homeostasis. We have previously cloned and characterised the *SbNHX1* gene from an extreme halophyte, *Salicornia brachiata*. Transgenic plants of *Jatropha curcas* with the *SbNHX1* gene were developed using microprojectile bombardment mediated transformation. Integration of the transgene was confirmed by PCR and Rt-PCR and the copy number was determined by real time qPCR. The present study of engineering salt tolerance in *Jatropha* is the first report to date. Salt tolerance of the transgenic lines JL2, JL8 and JL19 was confirmed by leaf senescence assay, chlorophyll estimation, plant growth, ion content, electrolyte leakage and malondialdehyde (MDA) content analysis. Transgenic lines showed better salt tolerance than WT up to 200 mM NaCl. Imparting salt tolerance to *Jatropha* using the *SbNHX1* gene may open up the possibility of cultivating it in marginal salty land, releasing arable land presently under *Jatropha* cultivation for agriculture purposes. Apart from this, transgenic *Jatropha* can be cultivated with brackish water, opening up the possibility of sustainable cultivation of this biofuel plant in salty coastal areas.

## Introduction

*Jatropha* is a second generation biofuel resource valued for its high oil content, low seed cost, land reclamation and easy adaptation to different kinds of marginal and semi marginal lands. The broad potential of this plant and multiple uses of different plant parts have made this species quite profitable for cultivation [1], [2]. As fossil fuels pose a great threat to energy security and also adversely affect the environment, initiatives are being taken for the partial replacement of fossil fuels by biofuels [3]. Recently, attention has been drawn to the high oil content (up to 50%) of its seeds that can be easily processed to partially or fully replace petroleum based diesel fuel [3], [4]. *Jatropha* is a non-food crop that separates this plant from fuel vs food controversy. Its oil properties include flash point 235°C and calorific value 39.63 MJ kg^−1^ that make this oil suitable as biofuel [1]. *Jatropha* oil contains 45.79% oleic acid (18∶1), 32.27% linoleic acid (18∶2), 13.37% palmitic acid (16∶0) and 5.43% stearic acid (18∶0) that is comparable with peanut, palm and corn oil [5]. *Jatropha* ranks next to oil palm in oil production per hectare, encouraging its cultivation worldwide [6]. There are concerns that *Jatropha* investors may drive its cultivation from marginal or degraded lands towards agricultural lands in order to reduce financial risk [7] but this possibility was recently ruled out [8].

*Jatropha* is well distributed in India [9]; this encourages its use as an alternative source for energy security in the country. India has a plan to expand biodiesel production and substitute 20% of diesel consumption by 2020 [10]. The land required to achieve this substitution target ranges from 4.24–66.98 million hectares (Mha) depending on the potential yield of the plant varieties and further its improvement programs. This target is feasible because of the extent of available wastelands in India [11]. CSIR-CSMCRI has been recognized worldwide and is actively working on *Jatropha* elite accessions’ selection, cultivation, genetic improvement and biodiesel production [6], [12].

Over 800 Mha of land throughout the world are affected by salt [13]. In India, the total arable land area is about 184 Mha, out of which 8.6 Mha is salt affected [14]. Salinity imposes various constraints on plant growth and yield from osmotic stress created due to high concentrations of Na^+^ and Cl^-^ ions [15]. There are some major effects of salt stress, *i.e.* water potential reduction, ionic imbalance or disturbances in ion homeostasis and ion toxicity, which inhibits enzymatic functions in key biological processes [15], [16].Salt tolerance depends on a range of physiological, biochemical and molecular adaptations activated by gene(s). The adaptive response to salinity is multigenic in nature, however a single gene can also increase the salt tolerance of a plant species [17], [18], [19]. Antiporters Na^+^/H^+^ are expressed by functional gene(s) and play an important role in plant salt tolerance. The basic strategy of salt tolerance is maintenance of Na^+^ homeostasis in the cytosol [15]; however it varies with the plant species. Ion homeostasis is carried out either by sequestration of excess sodium into the vacuoles via vacuolar Na^+^/H^+^ antiporters (NHX1) energized by the proton gradient generated by vacuolar membrane H^+^-ATPase and H^+^-pyrophosphatases [15], [17], or by active exclusion through Na^+^/H^+^ antiporters (SOS1) located on the plasma membrane [18], [19]. Therefore, the ability to maintain lower Na^+^ and Cl^-^ in the cytosol may be a key determinant of salt tolerance.

The previous study that vacuolar NHX proteins are capable of Na^+^ and H^+^ exchange across the tonoplast [15], [17], has been modified based on the biochemistry of the NHX proteins. Recently it was reported that NHX proteins do not discriminate between Na^+^ and K^+^ or have a preference for K^+^ transport [20], [21]. These reports suggest that NHX1 and NHX2 are vacuolar Na^+^–K^+^/H^+^ exchangers essential for active K^+^ uptake at the tonoplast, osmotic adjustment, turgor regulation, stomata function and cell expansion [22], [23].

*Jatropha* growth and yield are adversely affected by increasing salt concentration beyond 30–50 mM [24], [25], [26]. Therefore, *Jatropha* is considered to be a moderately salt-tolerant plant and its salinity tolerance is comparable with major crops such as soybean or wheat [27]. Development of transgenic *Jatropha* plants by the overexpression of selected salt-responsive gene(s) is a better choice for genetic improvement of the plant for enhanced salt tolerance and fastening the breeding of improved plants. There are several methods available for genetic transformation of plants but the widely used methods are the vector-mediated method using *Agrobacterium* and direct gene transfer via microprojectile bombardment. Both these methods have their advantages and limitations [28], [29]. We have reported an efficient microprojectile bombardment mediated transformation protocol for *Jatropha* [12] that has been used in this study.

**Figure 1:**
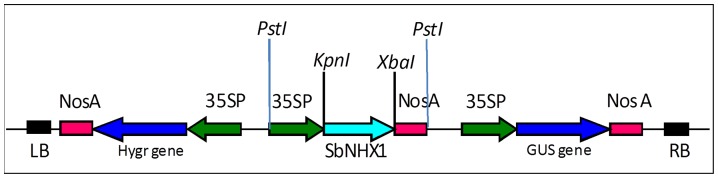
Schematic map of plant expression gene construct pCAMBIA1301-*SbNHX1*. Expression of *SbNHX1* is driven by the cauliflower mosaic virus 35S promoter.

Nevertheless, generating transgenic cultivars is not only limited by the success of the transformation process but also proper incorporation of the new stress tolerance. Recently, transgenic *Jatropha* plants were developed for trait improvement [30], [31]. Assessment of transgenic plants under salt stress conditions and understanding the physiological effects of inserted genes at the whole plant level remain as major challenges to overcome [32], [33]. A salt-tolerant *SbNHX1* gene (GenBank: EU448383) (Figure 1) encoding an active vacuolar Na^+^/H^+^ antiporter has been cloned and characterized from an extreme halophyte *Salicornia brachiata* in our laboratory [34]. The core objective of the present work is developing transgenic *Jatropha* with the *SbNHX1* gene for enhanced salinity tolerance. Salt tolerant transgenic *Jatropha* plants can be further utilized for sustainable cultivation in salt-affected marginal areas.

## Results

### Microprojectile Bombardment Mediated Transformation

**Figure 2:**
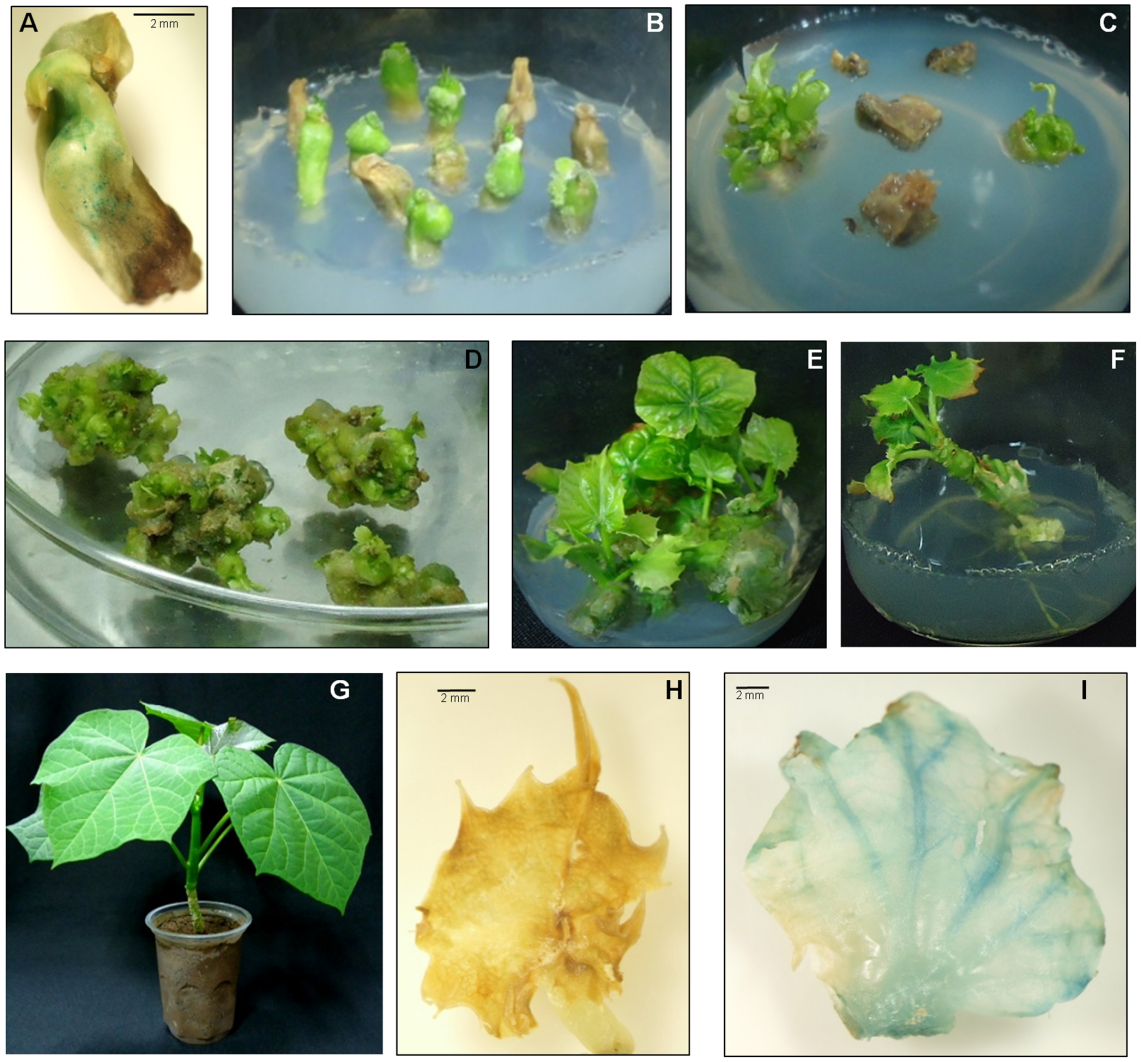
Genetic transformation of *J. curcas* via microprojectile bombardment. (A) Transient *gus* expression on embryo axis after 24 h of bombardment. Selection of transformants (B) during 1^st^ round (8 days old), (C) during 3^rd^ round (60 days old) on hygromycin. Regeneration of transformants; (D) multiple shoot induction, (E) multiple shoot regeneration (120 days old), (F) rooting (200 days old), (G) hardening of transgenic plant in plastic jar (250 days old). GUS assay of (H) non transformed leaf and (I) leaf of transgenic plant.

Five days pre-cultured embryo axes were bombarded at optimized parameters, *i.e.* 1 µm microcarrier size, 1100 and 1350 psi He pressure, with target distances of 9 and 12 cm, respectively [12]. After bombardment, 3–5 transformed embryos per shot per plate were selected randomly after 24 h and transient *gus* expression was assessed. Most of the explants showed discrete blue spots or regions (Figure 2a). Bombarded embryo axes were regenerated into transgenic plants after hygromycin selection (Figure 2b–f) as reported previously [12]. Putative transgenic plants with rooted shoots were transplanted into pots for hardening and acclimatization (Figure 2g and Table S1). Regenerated young leaves from putative transformed plants were assayed for constitutive expression of the *gus* gene using non-transformed leaves as control (Figure 2h–i).

### Molecular Analysis of Transgenic Plants

**Figure 3:**
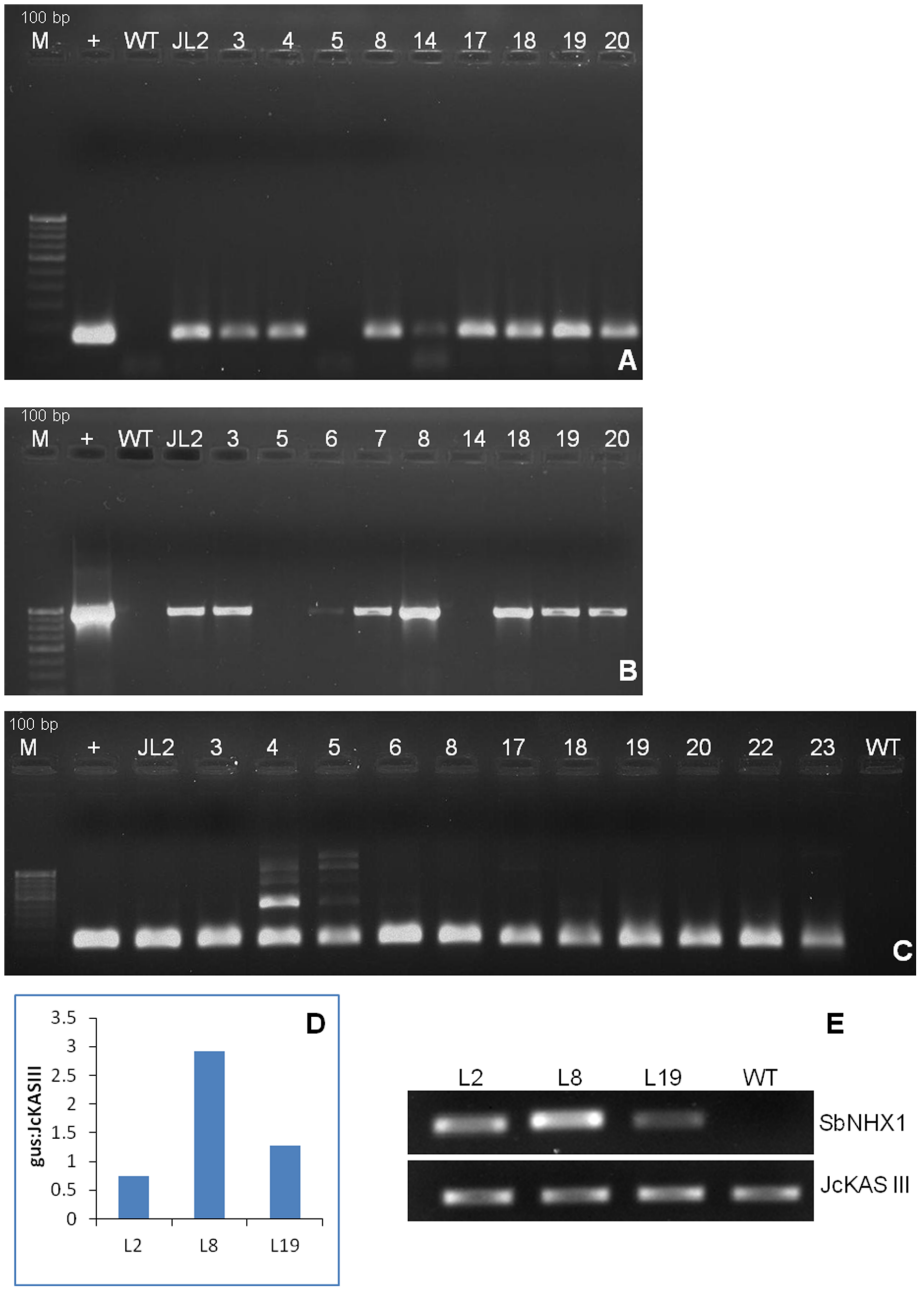
Molecular confirmation of transgenic Lines. PCR amplification of (A) *SbNHX1* gene (172 bp internal fragment), (B) *hptII* gene (963 bp) and (C) *gus* gene (157 bp internal fragment). Lane M is 100 bp Molecular weight marker ladder (Bangalore Genei, India); lane+is pCAMBIA1301-*SbNHX1* (positive control). (D) Real time qPCR analysis to determine the ratio of *gus* to *JcKASIII* gene in transgenic lines. Three lines L2, L8 and L19 have a ratio of 0.74, 2.92 and 1.27, respectively. (E) Reverse transcriptase PCR of SbNHX1 transgenic lines. Amplification was observed in transgenic lines while there was no amplification from WT; *JcKASIII* gene was used as an internal control.

Genetic transformation was confirmed by PCR amplification of 172 bp (internal fragment), 963 bp and 157 bp (internal fragment) of the *SbNHX1* gene, selectable marker *hptII* gene and reporter *gus* gene, respectively (Figure 3a–c). Real time quantitative PCR (RTqPCR) was performed to determine the copy number using a single copy *JcKASIII* gene as the internal control. Transgenic lines JL2, JL8 and JL19 showed *gus* to *JcKASIII* ratio 0.746, 2.922 and 1.274 respectively (Figure 3d). RTqPCR analysis revealed that transgenic lines JL2 and JL19 had single copy insertion while transgenic line JL8 showed a triple copy insertion. Melt curve analysis of the internal control *JcKASIII* gene and the transgenic lines individually showed single peaks that confirmed the absence of any dimer or other contamination. Single band amplification of the *gus* gene as well as the control gene was further confirmed by 1.2% agarose gel electrophoresis. The expression of the *SbNHX1* gene was further confirmed by reverse transcriptase PCR (Rt-PCR). Transgenic lines showed high expression of the gene whereas it was absent in WT plants (Figure 3e).

### Physiological and Biochemical Analysis

#### Leaf senescence assay, chlorophyll content and growth assay

**Figure 4:**
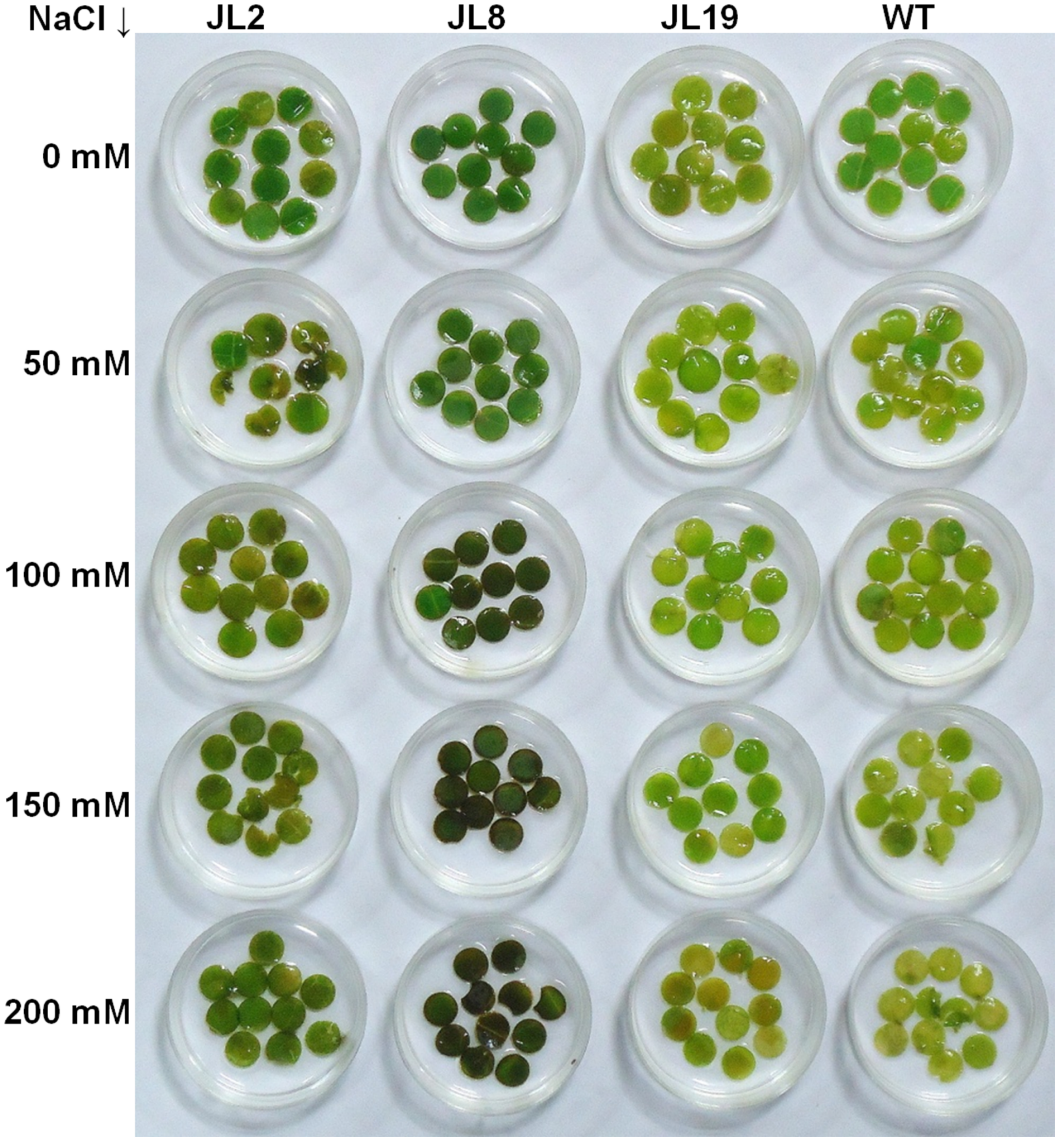
Leaf disc senescence assay of transgenic *J. curcas* for salt tolerance. Leaf discs of JL2, JL8 and JL19 transgenic lines and WT respectively were floated in 0 mM, 50 mM, 100 mM, 150 and 200 mM NaCl solution for 8 days.

**Figure 5:**
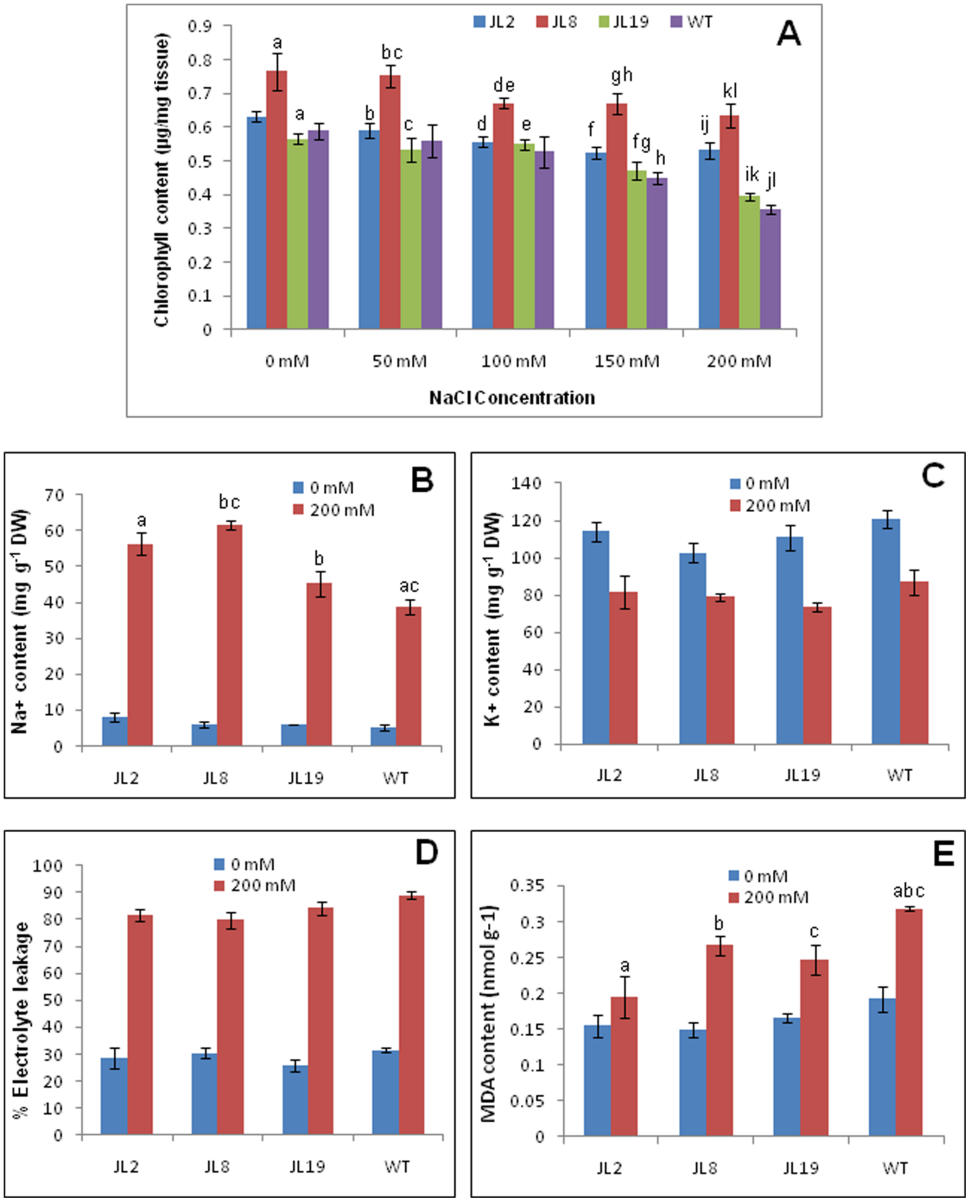
Physiological and biochemical analysis of transgenic lines. (A) Chlorophyll content assay of leaf of transgenic lines JL2, JL8, JL19 and WT at 0, 50, 100, 150 and 200 mM NaCl concentrations. (B) Na^+^ and (c) K^+^ ion content analysis; (D) Electrolyte leakage and (E) MDA content analysis of transgenic lines (JL2, JL8 and JL19) and WT at 0 and 200 mM of NaCl. Graph represents mean ± SD value and similar letters show significant difference at *P*<0.05.

**Figure 6:**
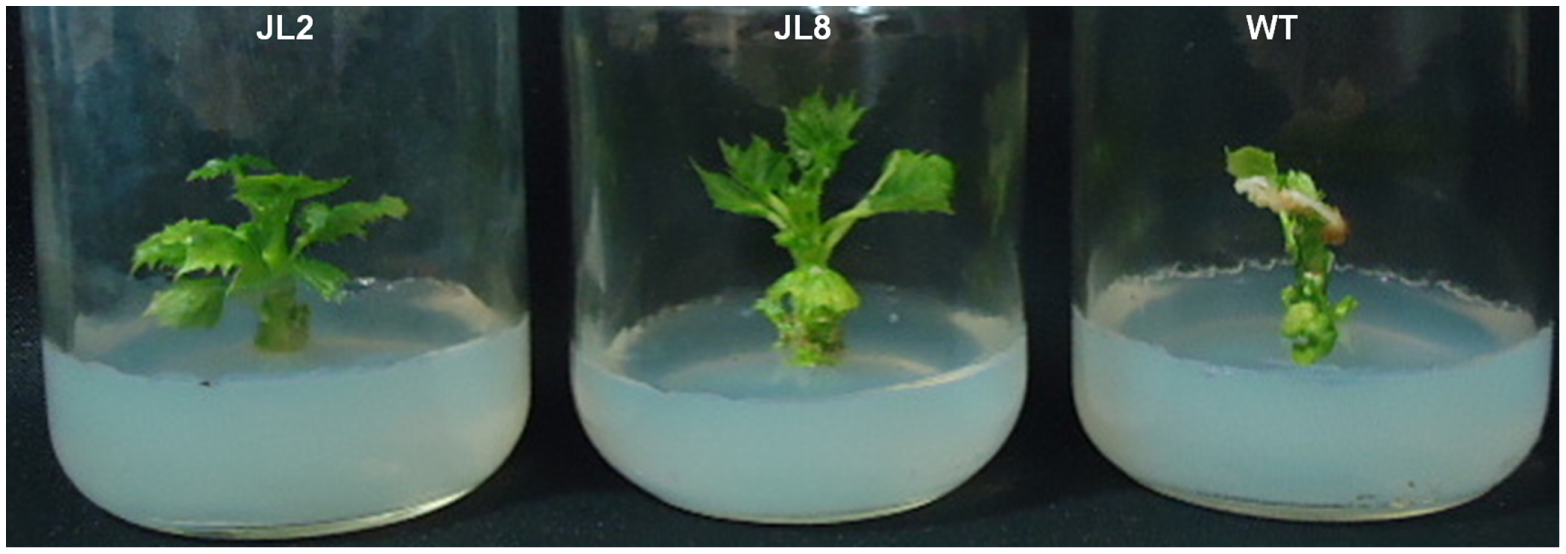
Effect of 200 mM NaCl on *in vitro* growing transgenic lines JL2 and JL8, and WT after 21 days.

The salt stress tolerance of T0 transgenic lines was studied by leaf disc senescence assay and chlorophyll estimation. Leaf discs from WT and T0 transgenic lines JL2, JL8 and JL19 were floated separately on 0, 50, 100, 150 and 200 mM NaCl for 8 days. Salinity-induced decreases in chlorophyll were lower in the *SbNHX1*-overexpressing lines compared to WT plants (Figure 4). The damage caused by salt stress was visualized by the degree of bleaching of leaf tissues. It was evident that the transgenic plants (JL2 and JL8) had a better ability to tolerate salinity stress. However, no significant difference in leaf senescence or chlorophyll content between WT and JL19 was observed. The chlorophyll content in the WT plants reduced significantly with increasing salt concentration while the transgenic lines (JL2 and JL8) retained higher amounts of chlorophyll than WT up to 200 mM NaCl (Figure 5a). Two months old transgenic lines and WT were transferred to SEM supplemented with 200 mM NaCl and kept for 21 days. Transgenic lines showed better growth, while WT showed bleaching in the leaves and growth was hindered (Figure 6).

#### Ion content analysis

At zero NaCl, transgenic lines and WT plants exhibited approximately similar Na^+^ content in leaf tissues. However, at 200 mM NaCl, there was significant increase in Na^+^ in all transgenic lines than in the WT plants (Figure 5b). These findings indicated that the transgenic lines accumulated more Na^+^ than the WT under the same stress conditions. In the case of K^+^, all the transgenic lines and WT showed non-significantly higher content at zero NaCl. However, at 200 mM NaCl, K^+^ decreased in both transgenic and WT plants. Although this decrease was also non-significant, WT exhibited higher K^+^ content than transgenic lines (Figure 5c). We also analysed the effect of NaCl on Ca^2+^ content (data not shown), but it was slightly and non-significantly increased at 200 mM NaCl. Transgenic lines showed slightly higher Ca^2+^ content than WT at 200 mM NaCl.

#### Electrolyte leakage and MDA content analysis

All the transgenic lines and WT exhibited approximately similar electrolyte leakage at zero NaCl while reduced electrolyte leakage was observed in transgenic lines during salt stress compared to WT but this decrease was non-significant (Figure 5d). Transgenic lines accumulated significantly less MDA than WT in response to salt stress, which was indicative of reduced oxidative damage in plants overexpressing *SbNHX1* (Figure 5e).

## Discussion

Plant tolerance to salt stress is a multigenic trait and requires the coordinated action of several genes, but it is evident from several reports that overexpression of a single gene can also impart salt tolerance to plants [17], [19], [35], [36]. Before proceeding to genetic transformation with any important gene and engage in further improvement strategies, optimization of an efficient regeneration and genetic transformation protocol is a crucial criterion to produce the required number of improved plants in a limited area. In the present study, we have successfully transformed *Jatropha* with the salt-responsive *SbNHX1* gene for its enhanced salt tolerance via microprojectile bombardment (Table S1). The optimized microprojectile bombardment protocol [12] was applied for bombardment of *Jatropha* embryo axes with the recombinant binary plasmid construct pCAMBIA1301-*SbNHX1*. RTqPCR is recently being used to determine copy numbers of integrated genes because of its high sensitivity and requirement for very small amounts of genomic DNA; this technique has been used in many recent reports [36], [37]. In our results, transgenic lines JL2 and JL19 showed single copy gene insertion while JL8 showed three copies. In the gene expression study by semi-quantitative Rt-PCR, JL8 showed higher expression than JL2 and JL19. The integration of three gene copies could be the possible reason for this higher expression.

In one of the two most recent reports on transgenic *Jatropha* for trait improvement, Qu *et al.* [30] developed marker-free transgenic plants with increase in high quality seed oil by down-regulating the seed-specific *JcFAD2-1* gene. In another report, transgenic *Jatropha* plants overproducing glycine betaine were developed for enhanced drought tolerance [31], but this study is at the primary level and needs to be studied further to obtain any concrete outcome. To date, several important plants have been transformed with *NHX1* gene cloned from different plant sources and its overexpression shown to enhance salt tolerance [35], [38].

Physiological analysis of transgenic plants represents the extent of expression of an integrated gene responsible for a trait. The strategy to accumulate Na^+^ inside vacuoles is used by many plants to survive under salt stress. An active vacuolar antiporter utilizes the proton motive force generated by vacuolar ATPases and pyrophosphatases to sequester excess Na^+^ into the vacuole [15]. SbNHX1 contains 11 strong transmembrane domains spanning in vacuolar membrane similar to some of the other halophytes and that actively regulates the ion homeostasis [34]. The response of the *SbNHX1*-transgenic lines JL2, JL8 and JL19 was assessed, and in a leaf senescence assay of T0 plants we studied the effect of NaCl on WT and transgenic lines in a dose-dependent manner (Figure 4). Transgenic lines showed better responses than WT, and chlorophyll content was found to be higher at 100, 150 and 200 mM NaCl concentrations after 8 days. However, at 50 mM NaCl there was no visible adverse effect on WT compared to transgenic lines and chlorophyll amount was on a par with that under conditions of zero NaCl (Figure 5a). Similarly, transgenic tobacco plants overexpressing *SbNHX1* showed better growth and chlorophyll content than WT plants at 100–300 mM NaCl concentrations [34]. Recently, Liu *et al.* [39] isolated *KcNHX1* and *KcNHX2* genes from the halophyte *Karelinia caspica* and showed that RNAi silencing of *KcNHX1* reduced the salt tolerance of *K. caspica* plants at 200 mM NaCl in a leaf disc assay, and chlorophyll content was decreased.In the ion content analysis, higher Na^+^ content and less K^+^ was observed in transgenic leaf tissues under salt stress. This can be attributed to ion homeostasis due to the activity of the vacuolar Na^+^/H^+^ antiporter SbNHX1. Significant increase in Na^+^ and reduction in K^+^ due to overexpression of AtNHX1 during salt stress has been reported in tomato [16], rapeseed [40], wheat [41], tall fescue [42] and buckwheat [43]. The growth of transgenic plants was not significantly affected by increased salinity, suggesting that K^+^ content was not compromised in the transgenic plants.

The transgenic lines (Jl2 and JL8) showed enhanced tolerance at 200 mM NaCl (Figure 6). The results show the positive effect of the Na^+^/H^+^ antiporter *SbNHX1* in T0 plants and their enhanced salt tolerance. The *PgNHX1* gene from *Pennisetum glaucum* has been reported to confer a high level of salinity tolerance when overexpressed in *Brassica juncea* [35] and tomato [38], and transgenic plants could withstand higher salt stress than WT in pot. Considering all the results, present study clearly demonstrate that overexpression of SbNHX1 improved the osmoregulatory capacity of transgenic lines by alleviating the toxic effect of Na^+^ due to its enhanced sequestration into the vacuole.

### Conclusions

In conclusion, we developed the functional transgenic *J. curcas* plants overexpressing *SbNHX1* and showing enhanced salt tolerance. Transgenic lines (JL2 and JL8) showed better response than WT up to 200 mM NaCl with respect to chlorophyll content, leaf senescence assay, ion content, electrolyte leakage and MDA content. The transgenic lines, JL2 and JL8 showed enhanced tolerance at 200 mM NaCl. To the best of our knowledge, this is the first report on enhanced salt tolerance in *Jatropha*. Salt tolerant transgenic *Jatropha* plants can be used for cultivation in salt-affected marginal areas for their sustainable management with biofuel production.

## Methods

### Plant Material and Culture Conditions

Mature decoated seeds of *Jatropha curcas* CP9 variety were surface sterilized with 2% (v/v) sodium hypochlorite for 15 min and washed 4–6 times with sterile distilled water. Embryo explants were dissected out from endosperm aseptically and pre-cultured for 5 days on MS (Murashige and Skoog) basal medium supplemented with 2.22 µM BAP (6-benzylaminopurine) +2.46 µM IBA (indole-3-butyric acid), 0.8% (w/v) agar and 3% (w/v) sucrose. All cultures were maintained under controlled laboratory conditions at 25±2°C under a 16/8 h light/dark photoperiod with a cool white fluorescent lamp with a light intensity of 35 µmol m^−2^ s^−1^.

### Plasmid Construct

The Binary plasmid vector pCAMBIA1301 (CAMBIA, Australia) containing the *SbNHX1* gene under the control of the CaMV35S promoter was named the pCAMBIA1301-*SbNHX1* gene construct (Figure 1) [34], which was used for microprojectile bombardment.

### Microprojectile Bombardment Mediated Transformation

Plasmid construct pCAMBIA1301-*SbNHX1* was isolated using a plasmid Miniprep kit (Qiagen, Germany) following the manufacturer’s protocol. Preparation of microcarriers, arrangement of explants and microprojectile bombardment conditions were the same as reported previously [12]. Bombardments were performed on 40–50 five days pre-cultured embryo axes per plate with a biolistic gene gun (PDS 1000/He, Bio-Rad).

### Selection and Regeneration of Transformants

After bombardment, the explants were kept in the dark at 25°C for 24 h and then transferred to shoot induction medium (SIM). Regeneration was carried out by following our previous reported protocol [12]. After 5–7 days, explants were transferred to selection medium (SIM) containing 5 mg/l hygromycin. For effective selection, the explants were transferred to shoot regeneration medium with increasing concentrations (6 and 7 mg/l) of hygromycin. After three cycles of selection, putative transformed shoots were transferred for approx. 40–60 days to shoot elongation medium for further shoot elongation.

For rooting, elongated shoots were transferred to root induction medium. After 4–6 weeks, plantlets with rooted shoots were transplanted into autoclaved red soil: sand: FYM (2∶2∶1) mix in small pots (5×10 cm) covered with transparent plastic lids and maintained under high humidity for 7–10 days, and thereafter gradually exposed to culture room conditions. Established plantlets were transferred to pots containing a mixture of red soil: sand: FYM (2∶2∶1) in green house conditions [12].

### Histochemical GUS Assay

Transient *gus* expression in embryo axis and leaves was assessed [44] after 24 h of transformation and randomly 3–5 explants were subjected to GUS assay for primary confirmation. Leaves of transgenic lines (4–5 months after transformation) were assayed for constitutive expression of the *gus* gene. GUS assay was done by incubating the tissues in freshly prepared GUS assay buffer (1 mg/ml X-Gluc with 0.05 M Na_2_HPO_4_, 0.5 mM K_3_Fe(CN)_6_, 0.5 mM K_4_Fe(CN)_6_, 10 mM EDTA and 0.1% (v/v) Triton X-100) for 12–16 h at 37°C in the dark. Thereafter tissues were destained with 70% alcohol to examine blue regions.

### PCR Amplification and Determination of Copy Number by Real Time Quantitative PCR

The genomic DNA of transformants was isolated and concentration was determined with a NanoDrop Spectrophotometer. Transformation was confirmed by PCR with *SbNHX1* gene specific (RT-NHX1F 5′-ATG GTG TTT GGG TTG CTG A-3′ and RT-NHX1R 5′-CTG CTT CGT CTT GGT TGT CC-3′); *gus* (reporter gene) specific (gusRTF: 5′-CGA CTG GGC AGA TGA ACA T-3′ and gusRTR: 5′-CTG TAA GTG CGC TTG CTG AG-3′) and *hptII* (hygromycin selection marker gene) specific (hptIIF: 5′-TTC TTT GCC CTC GGA CGA GTG-3′ and hptIIR: 5′-ACA GCG TCT CCG ACC TGA TG-3′) primers.

The genomic DNA was diluted to 1, 10 and 100 ng/µl concentrations and the real time quantitative PCR (RTqPCR) reaction was run in a Real Time iQ5 Cycler (Bio-Rad, USA). RTqPCR conditions were optimized for the *gus* gene primers (gusRTF and gusRTR) and *JcKASIII* gene (GenBank: DQ987701) primers (JcKAS1F: 5′-GCA CTT GGC TGC AAA ACA AAT-3′, JcKAS1R: 5′-CGT CCA GTC AAC ATA TCG AG-3′). The *JcKASIII* gene was used as an internal control because this is a single copy gene in the *Jatropha* genome [45]. The PCR reactions were carried out using 0.25 µM primers for both genes in a 20 µl reaction using the QuantiFast SYBR Green PCR reaction kit (Qiagen, USA). The following PCR conditions were maintained during RTqPCR: 95°C for 5 min (1 cycle), 94°C for 30 s, 60°C for 30 s, and 72°C for 45 s (40 cycles), 95°C for 1 min (1 cycle) and 60°C for 1 min (1 cycle). At the end of the PCR cycles, the products were subjected to melt curve analysis. The amplified product was run on a 1.5% agarose gel to confirm the expected size. The experiments were repeated twice independently with three replicates each time. Standard curves were plotted using threshold cycle (CT) value to determine reaction efficiencies [46] as follows:
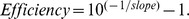



The efficiency values were put in the following equation [46] to determine the copy number ratio of *gus* to *JcKASIII*:
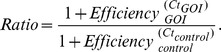



### Semiquantitative Rt-PCR Analysis

The total RNA was isolated from WT and transgenic plant samples using RNeasy Plus Mini Kit (Qiagen, Germany) and quantified with Nanodrop spectrophotometer (USA). The cDNA was prepared using 2 µg of total RNA using ImProm-II Reverse Transcription System kit (Promega, USA). Semiquantitative Reveres transcriptase-PCR analysis was performed using cDNA (1 µl) as template, *SbNHX1* gene as target and *JcKASIII* gene as an internal control using *SbNHX1* and *JcKASIII* specific primers (RT-NHX1F & RT-NHX1R; JcKAS1F & JcKAS1R). The PCR reactions were carried out in 1×PCR buffer supplemented with 200 µM dNTPs, 1.25 U Taq DNA polymerase and 5 pmol of each gene-specific primers with following conditions: an initial denaturation at 94°C for 3 min, 30 cycles at 94°C for 30 sec, 60°C for 30 sec and 72°C for 45 sec, followed by a final extension step at 72°C for 5 min. Rt-PCR experiments were repeated three times, and the amplification products were analysed with 1.2% agarose gel electrophoresis.

### Leaf Senescence Assay

Leaf disc assay was performed to analyse transgenic plants for their salt tolerance according to procedures described by Fan et al. [47]. Healthy leaves (third leaf from the top) from WT and transgenic plants (T0 generation) of similar age were detached. Leaf discs (10–12) 5 mm in diameter were punched out and floated in 5 ml sterilized distilled water with different concentrations of sodium chloride (0, 50, 100, 150 and 200 mM) for 8 days. The leaf discs were kept under 16 hours white light (35 µmol m^−2^ s^−1^)/8 hours dark at 25±2°C. The effects of this treatment on leaf discs were assessed by observing phenotypic changes. The experiment was repeated twice.

### Chlorophyll Estimation

Leaf discs of WT and the three transgenic lines, JL2, JL8 and JL19, treated with different concentrations of NaCl as described earlier were used for chlorophyll estimation. Treated leaf discs were homogenized thoroughly in 80% acetone and incubated for 1–2 h with shaking at 120 rpm in the dark. The homogenate was centrifuged at 3000 rpm for 5 min in the dark. The OD of the supernatant was taken at 645 and 665 nm, and chlorophyll was calculated per gram fresh weight of tissue [48].

### Effect of Salt on *in vitro* Growing Plants

Two months old *in vitro* growing WT and transgenic lines JL2 and JL8 were assessed for salt tolerance. Plants were grown in SEM supplemented with 200 mM NaCl and effect of salt was observed after 21 days.

### Ion Contents

For the analysis of ion contents, 0.5 g of fresh young leaves tissues were dried at 70°C for 48 h in an oven and dry weight was measured. The dried tissues were digested for 8–10 h with 4 ml perchloric acid and nitric acid solution (3∶1). Digested samples were dried on the hot plate, diluted to 25 ml with deionised water and filtered through 0.2-µm filter. Ion contents were measured by inductively coupled plasma optical emission spectrometer (Optima 2000DV, PerkinElmer, Germany).

### Electrolyte Leakage

Electrolyte leakage was measured according to Lutts et al. [49]. Leaf discs of similar size were collected from plants for each treatment and washed thoroughly with deionised water to remove surface-adhered electrolytes. The samples were kept in closed vials containing 10 ml of deionised water and incubated at 25°C on a rotary shaker for 24 h. Subsequently, the electrical conductivity (EC) of the solution (L_t_) was determined using conductivity meter (SevenEasy, Mettler Toledo AG 8603, Switzerland). Samples were autoclaved (120°C for 20 min), cooled up to 25°C and electrical conductivity (L_0_) was determined. The electrolyte leakage was defined as follows:
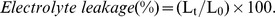



### Lipid Peroxidation

Lipid peroxidation was estimated by following the method described by Hodges et al. [50]. Leaf material (0.2 g) was homogenised in 5 ml of aqueous (80% v/v) acetone solution. In one set, 1 ml solution containing 0.65% (w/v) Thiobarbituric acid (TBA) and 20% (w/v) Trichloroacetic acid (TCA) was added to 1 ml extract, however in second set TBA was excluded. The mixtures were incubated at 90°C for 30 min and cooled to room temperature. Samples were centrifuged at 4,000 rpm for 3 min and the absorbance of the supernatants at 440 nm, 532 nm and 600 nm was determined. Concentration of malondialdehyde (MDA) produced by TBA reaction was determined according to the following equations:
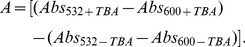
(1)

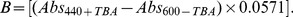
(2)

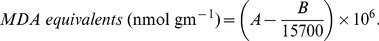
(3)


Each experiment was carried out in three replicates. To determine the significance of difference between the means of WT and transgenic plants of each treatment group, data was subjected to analysis of variance (ANOVA) and were expressed as mean ± SD. A Tukey HSD multiple comparison of mean test was used. When significant differences were found, *P*<0.05 was considered as significant. Significantly different mean values were indicated by similar letters.

## Supporting Information

Table S1 **Transformation efficiency and overall regeneration efficiency after microprojectile bombardment of embryo axes with pCAMBIA1301-** ***SbNHX1*** **gene construct.**
(DOC)
