# Correction: Developing Transgenic *Jatropha* Using the *SbNHX1* Gene from an Extreme Halophyte for Cultivation in Saline Wasteland

**DOI:** 10.1371/annotation/89bc2c6f-2799-4a5b-9f57-8e2fa3e14fc9

**Published:** 2013-12-10

**Authors:** Bhavanath Jha, Avinash Mishra, Anupama Jha, Mukul Joshi

The order of the authors is incorrect. The correct order of authors is as follows: Mukul Joshi, Anupama Jha, Avinash Mishra and Bhavanath Jha. The correct Citation is: Joshi M, Jha A, Mishra A, Jha B(2013) Developing Transgenic Jatropha Using the SbNHX1 Gene from an Extreme Halophyte for Cultivation in Saline Wasteland. PLoS ONE 8(8): e71136. doi:10.1371/journal.pone.0071136. 

