# Supplementary material for: Developing Transgenic Jatropha Using the SbNHX1 Gene from an Extreme Halophyte for Cultivation in Saline Wasteland
Source: PLoS One. 2013 Aug 5;8(8):e71136. doi: 10.1371/journal.pone.0071136 (PMC3733712; doi:10.1371/journal.pone.0071136)
Supplement: Table S1 — Transformation efficiency and overall regeneration efficiency after microprojectile bombardment of embryo axes with pCAMBIA1301- SbNHX1 gene construct. (DOC) [file pone.0071136.s001.doc]

**Table S1:** Transformation efficiency and overall regeneration efficiency after microprojectile bombardment of embryo axes with pCAMBIA1301-*SbNHX1* gene construct

| **S.N.** | **No. of embryos bombarded** | **After 3^rd^ selection** | **Transformation efficiency (%)** | **Regenerated transgenic plants** | **Regeneration efficiency (%)** |
| --- | --- | --- | --- | --- | --- |
| 1 | 40 | 3 | 7.5 | 0 | - |
| 2 | 60 | 15 | 25 | 0 | - |
| 3 | 125 | 46 | 36.8 | 2 | 1.6 |
| 4 | 100 | 35 | 35 | 2 | 2 |
| 5 | 80 | 21 | 26.25 | 2 | 2.5 |
| 6 | 50 | 19 | 38 | 0 | - |
| 7 | 150 | 58 | 38.66 | 3 | 2 |
| 8 | 100 | 38 | 38 | 1 | 1 |
| 9 | 60 | 24 | 40 | 0 | - |
| 10 | 100 | 43 | 43 | 2 | 2 |
| 11 | 90 | 38 | 42.22 | 1 | 1.11 |
| 12 | 80 | 29 | 36.25 | 0 | - |
| 13 | 90 | 36 | 40 | 1 | 1.11 |
| 14 | 100 | 37 | 37 | 1 | 1 |
| 15 | 100 | 41 | 41 | 2 | 2 |
| 16 | 100 | 39 | 39 | 2 | 2 |
| 17 | 100 | 42 | 42 | 7 | 7 |
| 18 | 100 | 43 | 43 | 2 | 2 |
| 19 | 250 | 98 | 39.2 | 7 | 2.8 |
| 20 | 200 | 87 | 43.5 | 9 | 4.5 |
| **Mean ± st. dev.** | | | **36.57 ± 8.41** |  | **2.308 ± 1.57** |

**Transformation efficiency:** Total plantlets regenerated after 3^rd^ round of hygromycin selection and confirmed by PCR or GUS assay.

**Regeneration efficiency:** Total plants established in green house after hardening.
